# Predatory interactions between prey affect patch selection by predators

**DOI:** 10.1007/s00265-017-2288-2

**Published:** 2017-03-16

**Authors:** Yasuyuki Choh, Maurice W. Sabelis, Arne Janssen

**Affiliations:** 1 Laboratory of Applied Entomology, Department of Horticulture, Chiba University, 648 Matsudo, Chiba, 271-8510 Japan; 2 Institute for Biodiversity and Ecosystem Dynamics, Department of Population Biology, University of Amsterdam, Amsterdam, The Netherlands

**Keywords:** Prey-prey interactions, Shared predator, Behaviour, Spider mites, *Neoseiulus californicus*, Thrips, Competition

## Abstract

**Abstract:**

When predators can use several prey species as food sources, they are known to select prey according to foraging efficiency and food quality. However, interactions between the prey species may also affect prey choice, and this has received limited attention. The effect of one such interaction, intraguild predation between prey, on patch selection by predators was studied here. The predatory mite *Neoseiulus californicus* preys on young larvae of the western flower thrips *Frankliniella occidentalis* and on all stages of the two-spotted spider mite *Tetranychus urticae*. The two prey species co-occur on several plant species, on which they compete for resources, and western flower thrips feed on eggs of the spider mites. A further complicating factor is that the thrips can also feed on the eggs of the predator. We found that performance of the predatory mite was highest on patches with spider mites, intermediate on patches with spider mites plus thrips larvae and lowest on patches with thrips larvae alone. Patch selection and oviposition preference of predators matched performance: predators preferred patches with spider mites over patches with spider mites plus thrips. Patches with thrips only were not significantly more attractive than empty patches. We also investigated the cues involved in patch selection and found that the attractiveness of patches with spider mites was significantly reduced by the presence of cues associated with killed spider mite eggs. This explains the reduced attractiveness of patches with both prey. Our results point at the importance of predatory interactions among prey species for patch selection by predators.

**Significance statement:**

Patch selection by predators is known to be affected by factors such as prey quality, the presence of competitors and predators, but little is known on the effects of interactions among prey species present on patch selection. In this paper, we show that patch selection by a predator is affected by such interactions, specifically by the feeding of one prey species on eggs of the other.

## Introduction

Early theory on prey patch selection assumed that predators select patches based on the rate of food intake (Charnov 1976; Stephens and Krebs 1986; Kacelnik et al. 1992), sometimes combined with the risk of predators becoming prey themselves (Sih 1980; Caraco et al. 1980). Since then, it has become clear that many more factors are involved in patch selection by predators, such as the need for a mixed diet (Belovsky 1978; Mayntz et al. 2005; Marques et al. 2015) and the avoidance of competing species (Janssen et al. 1995; Adler et al. 2001) and of intraguild predators (Moran and Hurd 1994; Magalhães et al. 2005; Choh et al. 2010). Also, when different prey species co-occur on the same patch, the interactions between these prey can affect patch quality and patch selection for predators (Werner and Peacor 2003; Ohgushi 2005; Schmitz et al. 2008). For example, populations of two herbivore species that feed on the same plant can affect each other through the induction of plant defences (Agrawal 2000; Viswanathan et al. 2005; Chung and Felton 2011), and these defences, in turn, may affect the quality of the herbivores as food for the predators (Havill and Raffa 2000; Harvey et al. 2003).

Another level of complexity is added to predator patch selection when the prey species involved do not have the same ecological role. For instance, one prey species (the so-called intraguild prey) may attack and feed on the other prey species (the so-called shared prey), whereas both are attacked by the predator (the intraguild predator, Holt and Polis 1997). Predators may avoid such patches because they have to compete for the shared prey with the intraguild prey. Interactions become even more complicated when one of the two prey species is an omnivore, i.e. feeds on the other prey species and on the shared host plant. In this case, the interaction between the two prey species can be classified as intraguild predation, but the omnivore and predators are also involved in intraguild predation. Furthermore, prey and omnivores may counter-attack the predators or even feed on them (Aoki et al. 1984; Barkai and McQuaid 1988; Palomares and Caro 1999). Hence, the interactions between two prey species can affect patch selection. Nevertheless, there are few studies that address the effect of combinations of several of these interactions. This is what we set out to do here.

**Fig. 1:**
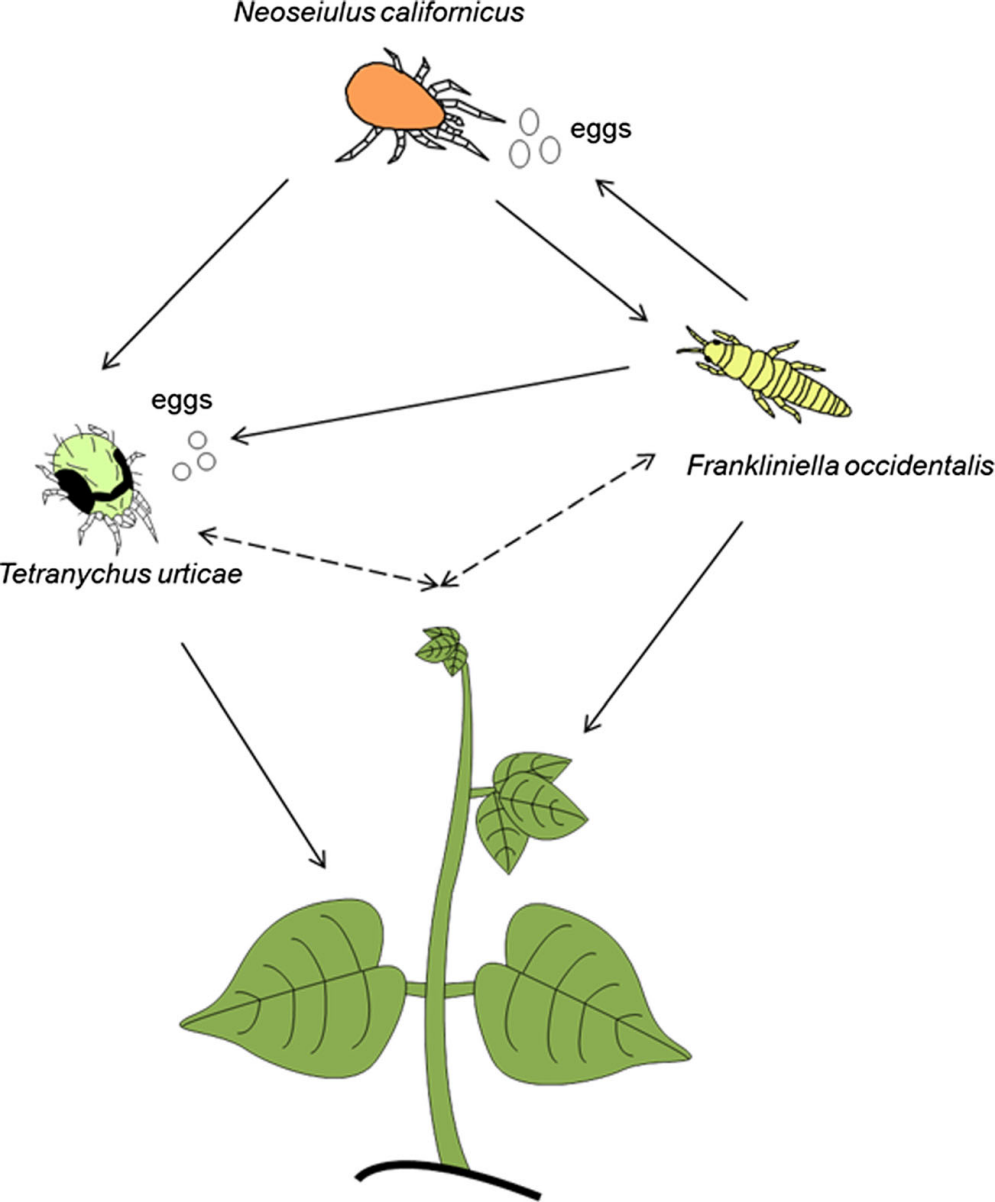
Interactions among plants, the two-spotted spider mite *T. urticae*, the western flower thrips *F. occidentalis* and the predatory mite *N. californicus*. Direct and indirect interactions are shown as *solid* and *dotted lines* respectively

We assessed the performance of a predator when feeding on each of two prey species separately and together and studied patch selection and the cues used for patch selection. The study system consisted of the omnivorous western flower thrips *Frankliniella occidentalis*, the phytophagous two-spotted spider mite *Tetranychus urticae* and the generalist predator *Neoseiulus californicus* (Fig. 1). The predator attacks all stages of the spider mite and mainly feeds on first-instar thrips larvae. Second-instar thrips larvae are largely invulnerable for predation by predatory mites (Bakker and Sabelis 1987; Belliure et al. 2008). The two prey species are both important agricultural pests and often co-occur. Although *N. californicus* performs better on spider mites than on thrips larvae (Walzer et al. 2004) and is known to be attracted to volatiles of plants attacked by spider mites (Janssen et al. 1990; Shimoda et al. 2005), nothing is known of its preference for either of the prey species or whether it performs better on a mixed diet of thrips and spider mites.

Western flower thrips are considered a pest of many plant species but also feed on eggs of the two-spotted spider mite and the predators (Fig. 1); hence, it is an intraguild predator of spider mites (Trichilo and Leigh 1986; Agrawal et al. 1999) and of predatory mites (Faraji et al. 2002; Janssen et al. 2002). In conclusion, many interactions known to affect predator patch selection occur in this simple food web, which can consequently be used to study the effect of combinations of these factors on patch selection by the predator.

Based on the information given above, we expected that the performance of the predators would depend on the stage of thrips larvae present, with higher performance on patches with first-instar larvae than on patches with second-instar larvae. In this paper, we considered patch selection at the spatial scale of a plant leaf. Because predatory mites feed and reproduce on the same patch, they were expected to select suitable patches for themselves and their offspring. We tested how the presence of the two prey species and the developmental stage of thrips larvae affected patch selection by the predatory mites. Subsequently, we investigated the cues involved in patch selection focusing on traces of thrips and spider mites and remains of spider mite eggs killed by thrips larvae.

## Materials and methods

Kidney bean plants (*Phaseolus vulgaris* cv. Nagauzura) were grown in soil in an incubator at 25 ± 2 °C and 50–70% relative humidity (r.h.), under a 16:8 h light/dark photoperiod. Plants were used 7–10 days after germination and had two fully expanded primary leaves and a trifoliate leaf.

Spider mites (*T. urticae*) were obtained from a culture maintained at the National Institute of Agrobiological Sciences in Tsukuba, Ibaraki, Japan, in 2010 and reared on kidney bean plants. Adult female spider mites, randomly selected from the culture, were used for experiments. Western flower thrips (*F. occidentalis*) were purchased from Sumika Techno Service Coporation (Takarazuka, Hyogo, Japan) in 2011 and reared on kidney bean plants. Predatory mites (*N. californicus*) were purchased from Arysta LifeScience (Tokyo, Japan). We reared the predatory mites on detached kidney bean leaves that were heavily infested with *T. urticae* and were added to the culture every other day. Cultures of the three arthropod species were maintained in incubators (25 ± 2 °C, 50–70% r.h., 16:8 h L/D), and individuals were randomly selected from the cultures to be used for the following experiments. All experiments were conducted in a climate-controlled room (25 ± 2 °C, 50–70% r.h., 16:8 L/D).

## Performance of predators on one or two prey species

We evaluated the net reproduction of predatory mites on each of the two prey species separately and together. To obtain patches with spider mites, 10 adult female spider mites were placed on a leaf disc (2.4-cm diameter) on water-saturated cotton wool in a Petri dish for 48 h, during which period they produced eggs and web. This spider mite web is an essential characteristic of spider mite patches, and *N. californicus* feeds on spider mites. For patches with thrips, another set of leaf discs received 10 first-instar thrips larvae; these patches were not incubated but used directly for the experiments to avoid the thrips larvae from growing and becoming less vulnerable for predation. To obtain leaf discs with both prey, leaf discs were first infested with 10 adult spider mites during 48 h as above, and subsequently, 10 first-instar thrips larvae were added to the discs, which were then immediately used for the experiment. An adult female predatory mite carrying an egg in her soma was introduced each on a separate disc. The numbers of predator offspring (i.e. eggs, larvae, nymphs and adults) were carefully counted under a binocular microscope 2 days after the release, corresponding to the vulnerable period of thrips larvae (Belliure et al. 2008). Each treatment had 23 replicates (i.e. predator individuals). A similar experiment was done with the exact same three treatments (i.e. only spider mites, spider mites with thrips and only thrips) but was evaluated in a similar manner 7 days after releasing the adult predators, at which time thrips larvae were largely invulnerable to predation (Belliure et al. 2008). The number of replicates was also 23 per treatment. The numbers of eggs and other stages of predators were compared with a generalized linear model (GLM) with a quasi-Poisson error distribution (log link), followed by an analysis of contrasts among treatments with the function glht of the package multcomp of R (Hothorn et al. 2008).

## Patch selection by predators

To test how the presence of two prey species affected patch selection by predators, we offered individual adult female predatory mites a choice between two connected leaf discs. We offered predators the following choices. (1) a leaf disc with spider mites plus their eggs and a leaf disc with first-instar thrips. (2) a leaf disc with first-instar thrips and a clean leaf disc. (3) a leaf disc with second-instar thrips and a clean leaf disc. (4) a disc with first-instar thrips and a disc with second-instar thrips. (5) a leaf disc with eggs and adult female spider mites plus second-instar thrips and a clean disc. (6) a disc with eggs and adult female spider mites plus second-instar thrips and a disc with eggs and adult female spider mites. (7) a disc with eggs and adult female spider mites plus second-instar thrips and a disc with second-instar thrips. Firstly, the two leaf discs (2 cm diameter) were placed on water-saturated cotton wool in a Petri dish (9 cm diameter, 2 cm depth). Subsequently, 10 adult female spider mites and/or 10 thrips larvae, either first or second instar, were introduced onto the respective leaf discs with a fine paint brush; spider mites were introduced 72 h before the experiment and were allowed to oviposit, hence, the leaf discs contained adult females, web and eggs. thrips larvae were introduced 24 h before the experiment. After this period of infestation, the two leaf discs were connected with a Parafilm bridge (4 cm long, 0.5 cm wide). Immediately after placing the bridge, a female predatory mite was introduced on the middle of it, and the position of the predator and her eggs was recorded 24 h after their release. The predators moved freely from disc to disc using the bridge (Y. Choh, pers. obs.). Hence, predators were expected to settle on their preferred patch after inspection of both patches. All predators and eggs were found on either of the leaf discs, not on the bridge. We repeated all tests 30 times. Data on patch choice were analysed with a binomial test to determine whether the distribution of predators over the two patches was significantly different from a 1:1 distribution. The numbers of predator eggs were compared between the two patches using Wilcoxon’s matched pairs signed-rank test. We also compared the total number of predator eggs (i.e. the sum of eggs on both discs) among choice tests with a GLM with a binomial Poisson error distribution (log link). Contrasts among tests were assessed with the general linear hypothesis test (function glht of the package multcomp, Hothorn et al. 2008).

## Cues used by predators for patch selection

A next step in our research was to investigate the cues that predators used in the patch selection experiments above, using similar leaf discs as above, also connected with a bridge. To obtain leaf discs with cues of both prey species, adult female spider mites were kept on leaf discs for 48 h, subsequently, thrips larvae were added to these discs with spider mites for another 24 h. Because this experiment focused on the role of prey-derived cues in patch selection by the predators, prey had to be prevented from moving from one patch to the other over the bridge, thus contaminating the other patch with cues. Therefore, only immobile prey, i.e. spider mite eggs, could be present during these experiments, and all adult spider mites and thrips larvae were removed from the discs. A sufficiently high number of spider mite eggs was left behind. To compensate for the decreased availability of food through removal of the mobile prey, we introduced more spider mites (30 adult females) than in the above experiments (10), thus, the arenas contained higher numbers of spider mite eggs than in the previous experiment. Besides these eggs, prey cues that were present on leaf discs were feeding damage, faeces and remains of spider mite eggs killed by thrips larvae. To specifically investigate the effect of these remains of killed spider mite eggs on patch choice by the predators, we divided leaf discs into two equal parts by placing a water-satiated cotton thread at the centre of the leaf discs. This cotton thread functions as barrier against movement of spider mites and thrips. Spider mites were allowed to feed and reproduce on one side of this barrier and thrips larvae on the other side. In this way, the leaf disc contained cues of spider mites and of thrips larvae, but no remains of spider mite eggs killed by the thrips. In these experiments, the other leaf disc was also divided into two and received adult female spider mites on one half and no thrips larvae on the other half. The discs were connected, an adult female predatory mite was released on the bridge, and data were collected as above.

Predatory mites were offered the following choices. (1) a leaf disc with cues and eggs of 30 spider mites and a leaf disc with cues and eggs of 30 spider mites plus cues of 10 second-instar thrips larvae and remains of spider mite eggs killed by the thrips larvae. (2) a leaf disc with cues and eggs of 30 spider mites and a leaf disc with cues and eggs of 30 spider mites plus cues of 10 first-instar thrips larvae (again including remains of killed eggs). (3) a leaf disc with cues and eggs of 30 spider mites plus cues of 10 second-instar thrips larvae plus remains of killed eggs and a leaf disc with cues and eggs of 30 spider mites plus cues of 27 first-instar thrips larvae and killed eggs (27 first-instar thrips larvae and 10 second-instar thrips larvae kill similar numbers of spider mite eggs, Y. Choh pers. obs.). (4) a leaf disc with cues and eggs of 30 spider mites and a leaf disc with cues and eggs of 30 spider mites plus cues of 10 second-instar thrips larvae, but without cues of killed spider mite eggs. (5) To verify whether the predators discriminated between leaf discs containing different numbers of spider mite eggs, we offered predators a choice between a leaf disc with cues and eggs of 30 spider mites (produced during 48 h) and a leaf disc with cues and eggs of 15 spider mites. (6) Finally, we offered predators the choice between a leaf disc with cues and eggs of 30 spider mites and a leaf disc with cues and eggs of 30 spider mites, of which 15 eggs (equivalent to the number of eggs killed by 10 second-instar thrips larvae, Y. Choh pers. obs.) were damaged with a fine needle. From the other leaf disc, 15 eggs were removed without damaging them, using a fine brush. This served to test for the effect of damaged spider mite eggs on patch selection by predators.

As above, data on patch choice were compared with a binomial test, numbers of predator eggs were compared with a Wilcoxon’s matched pairs signed-rank test and the total number of predator eggs with a GLM with a Poisson error distribution (log link). We furthermore compared results between different tests to verify the importance of cues for patch selection. For the choice of the predators, we used a contingency table analysis with a log-linear model (GLM with Poisson error distribution and log link Crawley 2013). To compare oviposition preferences, a GLM with a binomial error distribution (logit link) was used to test differences in the distribution of eggs over the two patches. Each experiment had 30 replicates.

## Results

### Reproduction of predators on one or two prey species

**Fig. 2:**
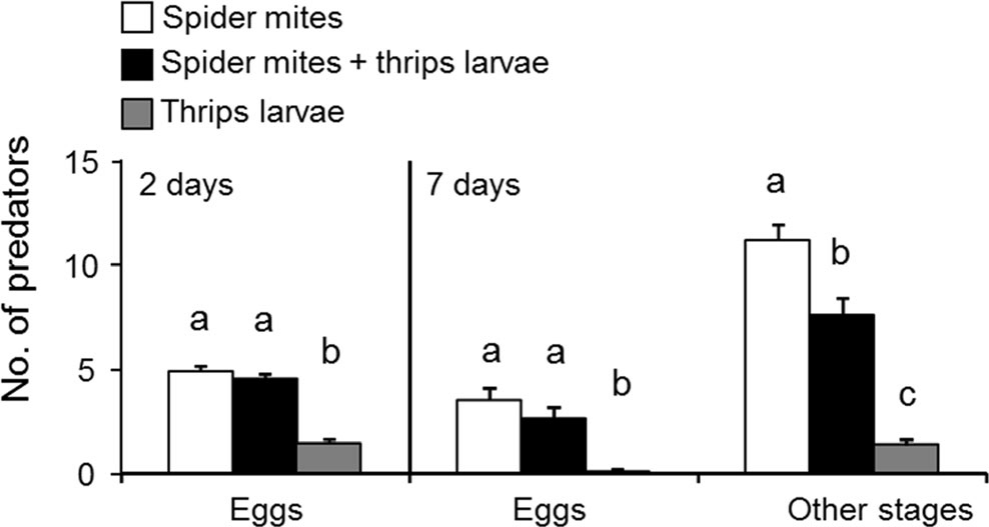
Reproduction of predators when being supplied with spider mites alone, thrips larvae alone and the two species together, during 2 and 7 days. *Different letters above the bars* indicate significant differences (*P* < 0.0001, GLM)

The numbers of predator eggs differed with the species composition of the prey, both after 2 and 7 days (GLM, 2 days, *F*_2,66_ = 57.8, *P* < 0.0001; 7 days, *F*_2,66_ = 22.05, *P* < 0.0001, Fig. 2). During the experiment lasting 2 days, 1.39 (SE 0.22) thrips larvae were killed, amounting to 0.70 thrips larvae per day. During 7 days, an average of 2.41 (SE 0.22) thrips larvae was killed, amounting to 0.34 thrips larvae per day. This difference in predation rate suggests that the thrips larvae became less vulnerable after 2 days. Fewer predator eggs were found with thrips larvae as the only food source than when spider mites alone or when the two prey species were offered as food (Fig. 2). The numbers of mobile predator stages after 7 days was also affected by the prey species offered (GLM, *F*_2,66_ = 68.8, *P* < 0.0001, Fig. 2). They were lowest on a diet of thrips larvae, higher on a mixed diet of thrips and spider mites and highest on a diet of spider mites (Fig. 2). These results suggest that: (1) irrespective of their age, thrips larvae are low-quality prey for the predators; (2) spider mites are of higher quality, resulting in the highest reproduction of the predators; and (3) the quality of a mixed diet of spider mites and thrips larvae depends on the age of the thrips larvae (Fig. 2). Based on these results, we expected the predators to prefer patches with spider mites and patches with spider mites plus young thrips larvae and not to prefer patches with only thrips larvae (either first or second instar) or patches with spider mites plus second-instar thrips. This was tested in the following experiments.

### Patch selection by predators

**Fig. 3:**
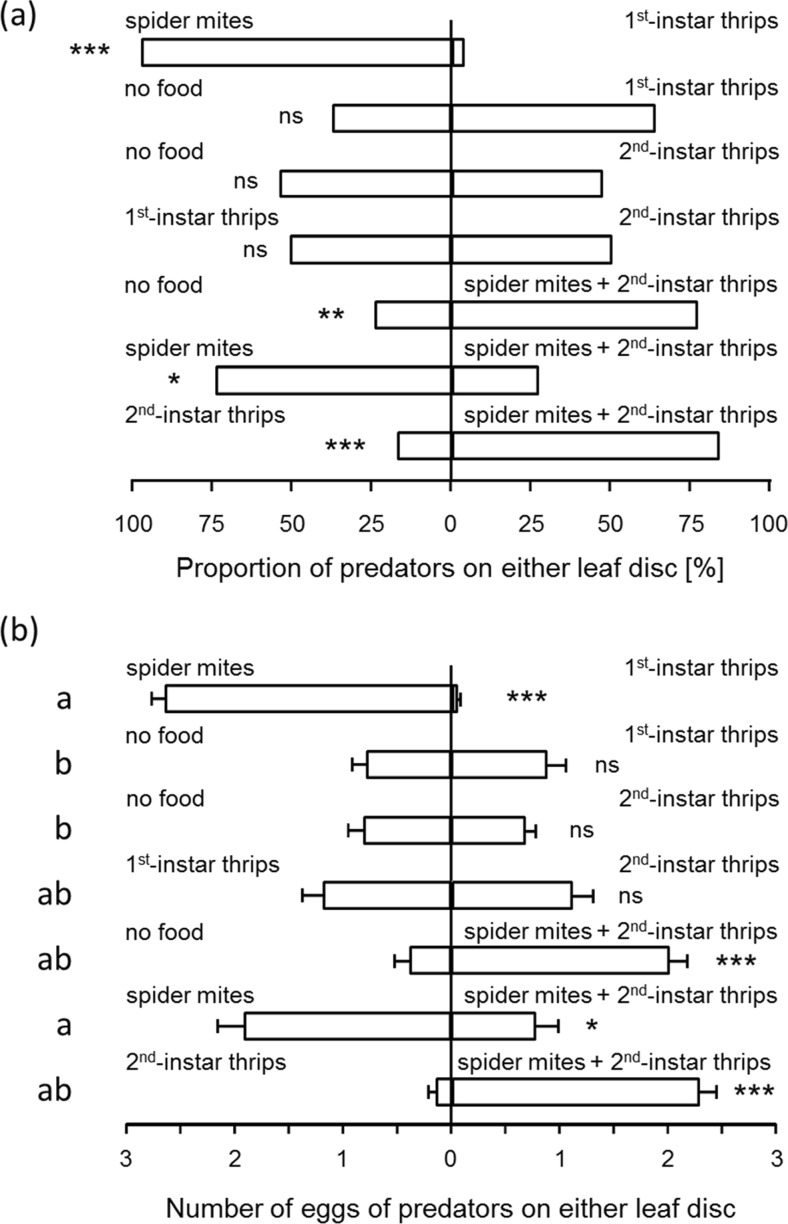
**a** Patch selection and **b** oviposition (+SEM) by predatory mites when offered a choice between the following two leaf discs: a disc with spider mites vs. a disc with first-instar thrips larvae (the *first bar*), an empty disc vs. a disc with first-instar thrips larvae (the *second bar*), an empty disc vs. a disc with second-instar thrips larvae (the *third bar*), a disc with first-instar thrips larvae vs. a disc with second-instar thrips larvae (the *fourth bar*), an empty disc vs. a disc with spider mites and second-instar thrips larvae (the *fifth bar*), a disc with spider mites vs. a disc with spider mites and second-instar thrips larvae (the *sixth bar*) and a disc with second-instar thrips larvae vs. a disc with spider mites and second-instar thrips larvae (the *seventh bar*). *Asterisks* indicate significance of the preference: *ns* not significant; *P < 0.05, **P < 0.01, ***P < 0.001 (binomial test for the adult predators (**a**) and Wilcoxon matched pairs signed-rank test for the eggs (**b**)). *Different letters next to the bars* indicate significant differences in the total numbers of eggs found on both discs among experiments (*P* < 0.0001, GLM)

Predators preferred leaf discs with spider mites to discs with first-instar thrips larvae (Fig. 3a, top bar), and more eggs were found on these leaf discs (Fig. 3b, top bar). When offered a choice between a leaf disc without food and a disc with first-instar thrips larvae, they did not show a preference for either of the two discs (Fig. 3a, b, second bars). A similar lack of preference was found with second-instar larvae versus clean discs (Fig. 3a, b, third bars). Because older thrips larvae are less vulnerable and kill more spider mite eggs than younger ones (Belliure et al. 2008; Y. Choh pers. obs.), we expected the predators to prefer leaf discs with first-instar thrips larvae to leaf discs with second-instar thrips larvae. Contrary to expectation, the predators did not show such preference (Fig. 3a, b, fourth bars). Leaf discs with spider mites plus second-instar thrips larvae were significantly more attractive to predators than leaf discs without food (Fig. 3a, b, fifth bars). Predators preferred leaf discs with spider mites to leaf discs with spider mites plus second-instar thrips larvae (Fig. 3a, b, sixth bars), showing that the presence of thrips larvae reduced the attractiveness of leaf discs with spider mites. Finally, leaf discs with spider mites plus second-instar thrips larvae were more attractive than leaf discs with only second-instar thrips larvae (Fig. 3a, b, lower bars), showing that the presence of spider mites increased the attractiveness of leaf discs with thrips larvae. The total number of predator eggs (adding the eggs of both discs) produced differed significantly among experiments (Fig. 3b, GLM, Chi^2^ = 19.6, d.f. = 6, *P* = 0.003). Most predator eggs were found when one of the leaf discs contained only spider mites, least when only thrips were present on one disc and the other was empty (Fig. 3b), confirming the results of the performance experiment (Fig. 2). The results also suggest that total egg production by predators was affected by prey species on two connected patches.

### Cues used by predators for patch selection

**Fig. 4:**
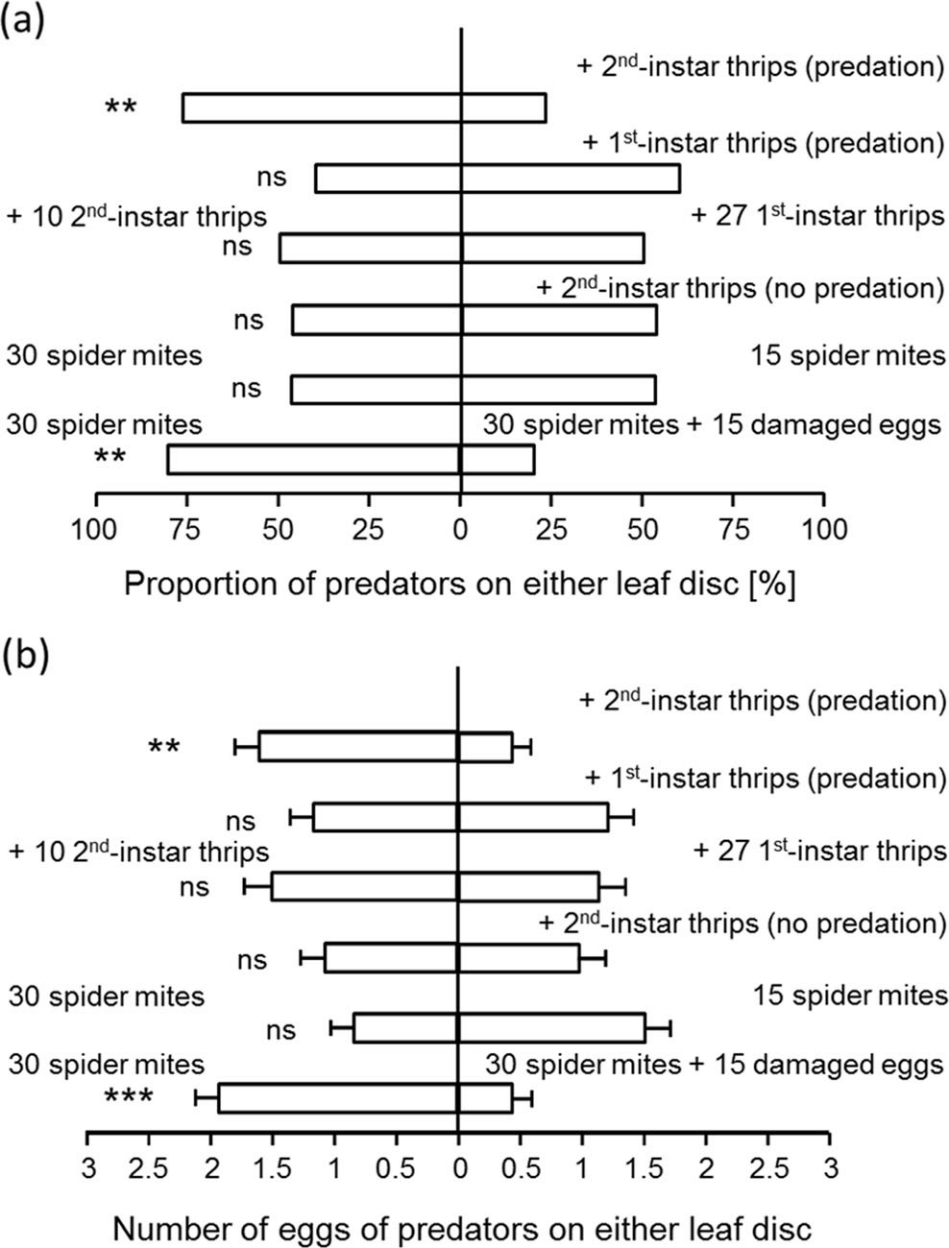
**a** Patch selection and **b** oviposition by predatory mites when offered two leaf discs. **a** Adult female patch selection, showing the proportion of adult female predators found on each of the two discs. **b** Oviposition preference, showing the numbers of eggs produced on each leaf disc (+SEM). Each disc contained eggs and cues of spider mites. In addition, one of the leaf discs contained cues of 10 second-instar thrips larvae (*first bars from above*) or cues of 10 first-instar thrips larvae (*second bars*). The *third bars* concern a choice between leaf discs (both with spider mite eggs and cues), of which one contained additional cues of 10 second-instar thrips larvae and with the other with additional cues of 27 first-instar thrips larvae. The *fourth bars* refer to an experiment where spider mites and thrips produced cues each on a separate half of one of the leaf discs so that thrips could not prey on spider mite eggs. This disc was offered together with a disc with only spider mite eggs and cues. The *fifth bars* concern a choice between a disc with eggs and cues produced by 30 adult female spider mites and a disc with eggs and cues of 15 spider mites. The *last bars* show the choice of predators between a disc with eggs and cues of 30 spider mites and a similar disc, on which eggs were damaged to mimic egg predation. See legend to Fig. 3 for details of statistics. *Asterisks* indicate significance of the preference: *ns* not significant; **P < 0.01, ***P < 0.001

When predators were offered a choice between one disc with cues and eggs from 30 spider mites and a disc with cues and eggs of spider mites plus cues of second-instar thrips larvae and remains of killed spider mite eggs, predators were mainly found on the disc without thrips cues (Fig. 4a, top bar) and also produced more eggs there (Fig. 4b, top bar). This shows that the cues associated with second-instar thrips larvae (including remains of spider mite eggs killed by the thrips) rendered the patch less attractive. When offered a choice between two similar discs as above, but with one disc with cues of first-instar thrips larvae and remains of spider mite eggs, no such preference was observed (Fig. 4a, b, second bars from above), suggesting that the presence of cues associated with first-instar thrips larvae, including remains of eggs, did not significantly affect patch choice. This is confirmed by comparing the preference of these two choice experiments, which shows that they were significantly different (adult predators: cf. top 2 bars of Fig. 4a, GLM, Chi^2^ = 8.53, d.f. = 1, *P* = 0.0035; eggs: cf. top 2 bars of Fig. 4b, *F*_1,58_ = 7.28, *P* = 0.009). This difference might have been caused by differences in the cues of first-instar versus second-instar larvae or by differences in the amount of remains of spider mite eggs. We therefore subsequently offered predators a choice between a leaf disc with cues and eggs of spider mites plus cues of 27 first-instar thrips larvae and a disc with cues and eggs of spider mites plus cues of 10 second-instar thrips, thus equalizing the amount of remains of killed spider mite eggs on both sides. Predators now showed no significant preference (Fig. 4a, b, third bars). This suggests that predators mainly responded to the amount of remains of spider mite eggs rather than to specific cues of first-instar or second-instar thrips larvae.

To further test the effect of remains of preyed spider mite eggs, we offered predators a choice between a disc with cues and eggs of spider mites and of second-instar thrips larvae but without remains of spider mite eggs and a disc with cues and eggs of spider mites but without cues of thrips, and the predators indeed did not show a significant preference (Fig. 4a, b fourth bar). These results differed significantly from those with cues of spider mites plus thrips plus remains of spider mite eggs (cf. Fig. 4a, first and fourth bars, Chi^2^ = 5.83, d.f. = 1, *P* = 0.016; Fig. 4b, first and fourth bars, *F*_1,58_ = 4.86, *P* = 0.031). Because predation of spider mite eggs by thrips also reduced the number of spider mite eggs on the discs, we subsequently offered predators a choice between leaf discs with different densities of eggs and cues of spider mites. This did not result in a significant preference of the predators (Fig. 4a, b, fifth bars). Finally, the presence of artificially damaged spider mite eggs significantly reduced the attractiveness of a patch (Fig. 4a, b, lower bars).

There were no significant differences in the total number of eggs produced by predators in the various experiments (cf. total oviposition in Fig. 4b, GLM, Chi^2^ = 3.45, d.f. = 6, *P* = 0.75).

## Discussion

### Reproduction and patch selection by predators

The predatory mites performed best when feeding on spider mites and worst when feeding on thrips larvae (Fig. 2). The low performance of predators on thrips larvae alone might have been caused by thrips larvae being an inferior food for the predators, but also because thrips are known to kill eggs of predatory mites (Janssen et al. 2002, 2003; Magalhães et al. 2005). During the first 2 days, the performance of predators on patches with both prey species did not differ significantly from that on patches with spider mites only (Fig. 2). Possibly, young thrips larvae killed few eggs of the predators, and the performance of the predators was not affected by feeding on a mixed diet of spider mite eggs and thrips larvae. Alternatively, it is possible that the mixed diet did result in higher egg production by the predators, but that thrips larvae killed part of the eggs. After 7 days, the predators performed less well on patches with both prey species than on patches with spider mites only (Fig. 2). During most of this period, the thrips larvae were less vulnerable and probably killed eggs of the predators, resulting in overall fewer offspring. They also killed spider mite eggs, which will have resulted in a lower density of these eggs, the superior food source for the predators, which may then have resulted in lower performance of the predators. However, there were still a number of spider mite adults and eggs left on the leaf discs at the end of the performance experiment, suggesting that there was no shortage of the superior food source. It is also possible that the presence of thrips larvae induced egg retention in the predators (Faraji et al. 2001; Montserrat et al. 2007; de Almeida and Janssen 2013).

The patch preference of the predators largely coincided with their performance: They preferred patches with spider mites and spider mites plus thrips larvae over patches without food (Fig. 3). Surprisingly, patches with first-instar thrips larvae were not attractive, although the predators can feed on them. In contrast, second-instar thrips larvae are much less vulnerable to attacks by predatory mites (Bakker and Sabelis 1987; Belliure et al. 2008), and patches with this prey type were therefore expected to be unattractive. Nevertheless, the predators showed a similar response to patches with first-instar and second-instar thrips larvae.

Probably, thrips larvae also killed predator eggs on patches containing spider mites plus thrips but possibly killed fewer predator eggs on patches that also contained spider mites (Fig. 2) because they preferred eggs of the latter. Predator eggs may also have ran a lower risk of being killed by thrips because of the protection offered by spider mite web (Roda et al. 2000; Lemos et al. 2015). Both potential, not mutually exclusive, explanations would result in higher numbers of predator offspring on patches with spider mites plus thrips than on patches with only thrips, which was indeed what we found (Figs. 2 and 3b). Further experiments are needed to identify which of these mechanisms play a role.

### Cues used by predators for patch selection

Another question addressed here concerns the nature of the cues used by the predators when selecting patches. Several types of cues could potentially be involved in patch selection by *N. californicus*. First, the volatile cues emanating from leaf discs damaged by spider mites may attract the predators (Janssen et al. 1990; Shimoda et al. 2005). Predators and parasitoids are known to respond to plant volatiles that are produced upon herbivore feeding (Sabelis and van de Baan 1983; Turlings et al. 1990; Dicke et al. 1990) but can also use volatile cues to avoid plants with prey and competitors (Janssen et al. 1997). Our data suggest that volatiles emanating from leaf discs attacked by thrips were not attractive (Figs. 3 and 4), although it cannot be ruled out that predators were initially attracted by such volatiles and subsequently left the patches upon contact with non-volatile cues associated with thrips larvae. These latter cues are the second type that potentially affects patch choice by the predators. Contact with such non-volatile prey cues or with prey themselves may have been involved in patch choice. For example, the web produced by spider mites is known to arrest predatory mites (Schmidt 1976; Hislop and Prokopy 1981). Although the spider mite web was largely destroyed when investigating the cues used by predators, some web will have remained on the leaf discs that had been infested with spider mites. The cues left by thrips larvae seem to be neither attractive nor repellent (Fig. 4). In fact, there are several lines of evidence that patch choice by the predators was strongly affected by cues associated with predation of spider mite eggs by thrips larvae, i.e. remains of killed spider mite eggs. First, predators had a lower preference for leaf discs on which second-instar thrips larvae had killed eggs of spider mites than for discs on which no eggs had been killed but did not show such a decreased preference when the thrips larvae were prevented from killing spider mite eggs (Fig. 4). Second, first-instar thrips larvae kill fewer spider mite eggs than do second-instar larvae, and patches with remains of spider mite eggs killed by first-instar thrips larvae were not significantly less attractive than patches without such remains. Third, when compensating for lower predation of spider mite eggs by first-instar thrips larvae through increasing the numbers of larvae, we indeed no longer found significant differences in attractiveness between patches with first-instar and second-instar thrips larvae, suggesting that cues associated with killing spider mite eggs were involved in patch selection (Fig. 4). Fourth, the decreased preference of predators for patches with experimentally killed spider mite eggs further confirmed this (Fig. 4).

We therefore conclude that cues associated with thrips larvae do not seem to be decisive in patch selection by *N. californicus* but that cues associated with killed spider mite eggs resulted in decreased preference of this predator. This is in contrast with earlier findings with the predatory mite *Phytoseiulus persimilis*, which was attracted by odours associated with predation on spider mite eggs by conspecifics (Janssen et al. 1997). In predator-prey systems, prey are known to exhibit antipredator responses to cues related to predation on heterospecific prey (Shriner 1998; Fraker 2009; Elvidge and Brown 2015). These studies suggest that cues of killed prey are associated with predation risk for heterospecific prey. In our system, cues associated with killed spider mite eggs can inform predators of the presence of both competitors and intraguild predators (i.e., thrips). By responding to such cues, the predators might avoid competition for food and intraguild predation.

We expected that the predators would discriminate between patches with first-instar thrips larvae, which are prey but also kill predator eggs, and patches with second-instar larvae, which are much more difficult to prey upon and also kill predator eggs. However, we found no evidence for this (Fig. 3a). It thus seems that the predators do not discriminate between the two stages of thrips larvae. Possibly, first-instar and second-instar thrips larvae always co-occur on plants that are naturally infested by thrips, hence are not spatially separated. Thus, predators may not have been selected for discriminating between patches with first-instar or second-instar thrips larvae. The only way in which the predators do seem to discriminate between patches with first-instar and second-instar thrips larvae seems to be indirectly, i.e. through the numbers of spider mite eggs killed by the larvae. It is likely that the predators would further use cues of own or conspecific eggs killed by the thrips larvae (Faraji et al. 2001; Janssen et al. 2002). This needs further research.

It is well known that predators have density-mediated and trait-mediated indirect effects on communities of prey and non-prey (Abrams 1995; Schmitz 1998; Werner and Peacor 2003). These studies suggest that predators affect interactions among prey and non-prey species. Here, we show the contrary: the behaviour of predators was affected by interactions between two prey species (Toscano et al. 2010), specifically by a predatory interaction between the two prey species. The two prey species studied here can affect each other not only through changes in food quality but also through intraguild predation of thrips larvae on spider mite eggs. This intraguild predation is also affected by food quality: when plant quality is low, thrips larvae are known to increase predation on eggs of spider mites (Agrawal et al. 1999; Agrawal and Klein 2000) and on predator eggs (Janssen et al. 2003). In addition, the strength of egg predation depends on developmental stages of thrips. As a result, the quality of a prey patch for predators will change with the development of thrips larvae on that patch. We suggest that such interactions among prey species are not rare in nature. For example, Polis (1991) gives several examples of predators that feed on prey and on the prey of the prey, whereas the two prey species consume similar resources. In fact, such interactions always occur when two prey species are involved in intraguild predation and have a shared predator. Intraguild predation occurs at all trophic levels except for the lowest one (Arim and Marquet 2004), and interactions between prey as described here will be predominantly expected when the two prey are of intermediate trophic levels (for example, herbivores and omnivores) and share a predator. We therefore expect that interactions among prey often affect patch selection by predators in other predator-prey systems. Thus, not only the prey quality but also the interactions among prey are important for predator behaviour, in particular patch selection.
